# Field Study on the Immunological Response and Protective Effect of a Licensed Autogenous Vaccine to Control *Streptococcus suis* Infections in Post-Weaned Piglets

**DOI:** 10.3390/vaccines8030384

**Published:** 2020-07-14

**Authors:** Lorelei Corsaut, Marty Misener, Paisley Canning, Guy Beauchamp, Marcelo Gottschalk, Mariela Segura

**Affiliations:** 1 Research Group on Infectious Diseases in Production Animals (GREMIP) and Swine and Poultry Infectious Diseases Research Centre (CRIPA), Faculty of Veterinary Medicine, University of Montreal, Saint-Hyacinthe, QC J2S 2M2, Canada; lorelei.corsaut@umontreal.ca (L.C.); marcelo.gottschalk@umontreal.ca (M.G.); 2 South West Ontario Veterinary Services, Stratford, ON N4Z 1H3, Canada; mmisener@Southwestvets.ca (M.M.); pcanning@southwestvets.ca (P.C.); 3 Biostatistics Office, Faculty of Veterinary Medicine, University of Montreal, Saint-Hyacinthe, QC J2S 2M2, Canada; guy.beauchamp@umontreal.ca

**Keywords:** *Streptococcus suis*, infection, field, autogenous bacterin, vaccine, pigs

## Abstract

*Streptococcus suis* is one of the most important bacterial pathogens in weaned piglets and responsible for serious economic losses to the swine industry. Currently, mostly autogenous vaccines composed of killed bacteria (bacterins) are available. However, immunological and protective data from field studies are missing. We report for the first time a comparative field study on the immunological response induced by an autogenous vaccine applied to either piglets or sows in a farm with recurrent *S. suis* problems. (I) Piglets from non-vaccinated sows received an autogenous bacterin during the first week and at three weeks of age. (II) Sows received the vaccine at five and three weeks pre-farrowing and piglets were non-vaccinated. Levels, isotype profile and opsonophagocytosis capacity of the serum antibodies induced by vaccination were evaluated. Vaccination of piglets failed to induce an active immune response. Vaccination of sows induced a significant increase in anti-*S. suis* antibodies, mainly composed of IgG1. However, isotype switching was modulated by the *S. suis* serotype included in the vaccine formulation. Despite this antibody increase in vaccinated sows, transfer of maternal immunity to piglets was not different from the control group (i.e., piglets from non-vaccinated sows). Notably, levels of maternal antibodies in piglets were already very high with marked opsonophagocytosis capacity at one week of age, independently of the vaccination program. However, their levels decreased by three weeks of age, indicating possible absence of antibodies in the post-weaning high-risk period. These observations correlated with lack of clinical protection in the farm. Overall, a piglet or a sow vaccination program herein mostly failed to induce lasting protection in nursery piglets. An improvement of vaccine formulation or an optimized program may be required.

## 1. Introduction

*Streptococcus suis* is responsible for important economic losses to the porcine industry worldwide [1]. It is also one of the most important complications of infections caused by the porcine reproductive and respiratory syndrome virus (PRRSV) [1]. Clinical features associated with *S. suis* infection in pigs are meningitis, arthritis, endocarditis, polyserositis and septicemia with sudden death. *S. suis* is an encapsulated bacterium and the antigenic diversity of the capsular polysaccharide (CPS) is the basis of bacterial classification into 29 serotypes [2]. Usually, few virulent serotypes (mostly serotypes 2 and 9) are recovered from diseased animals in Europe [3,4]. However, the situation is much complicated in North America, where many serotypes are routinely isolated from diseased pigs, without a clear predominance of a single serotype across the countries [5,6]. In addition, *S. suis* has been reported to be an emerging zoonotic pathogen with the greatest risk for people who have close contact with pigs or unprocessed pork such as, in Western countries, pig farmers and workers, butchers, meat inspectors and swine veterinarians.

As the complexity of *S. suis* epidemiology in swine increases (multiple strains, multiple serotypes), field reports describing difficulties in disease control and management are common [7]. Early medicated weaning and segregated early weaning practices do not eliminate *S. suis* infection, since piglets are infected very early in life or even during farrowing [1]. Indeed, control of *S. suis* disease is frustrating. Antibiotics can prevent clinical outbreaks, but those that have efficacy are products that the industry is trying to use as little as possible, given their importance in both human and veterinary medicine [1]. Recent data on antimicrobial susceptibility of *S. suis* are alarming [8]. Very high rates of resistance to macrolides/lincosamides and tetracyclines are observed and attributed to the intensive use of antimicrobials in pigs [8]. Indeed, *S. suis* is considered a reservoir for antibiotic resistance and it represents a high risk of transmission of such resistance to other veterinary and human pathogens due to the presence of mobile genetic elements carrying resistance genes transferable at high frequency within the species and, even more alarming, between bacterial species [9].

Prevention of *S. suis* disease should be concentrated on management of predisposing factors and, mainly, vaccination. Universal efficacious commercial vaccines could not be developed so far, probably due to the presence of a high number of serotypes (with no cross-protection against each other) and a high variation of strains within a given serotype [7,10]. The reduction in the use of antimicrobials and the presence of a high variety of serotypes, led to an increased popularity of autogenous vaccines. These vaccines are bacterins (whole cell killed bacteria with a complex antigen charge) based on the predominant strain(s) recovered from diseased pigs in the affected farm and produced by accredited laboratories. Most studies evaluating the protective capacity of bacterins have been carried out under laboratory conditions with vaccines produced with reference strains and presented contradictory results [7,11]. The limited protective response obtained with experimental bacterins has been attributed to failure of the whole-bacterial antigens to elicit an immune response due to loss of antigenicity caused by the killing procedure, to production of antibodies to antigens not associated with protection and/or the use of inappropriate adjuvants [7,11]. However, the exact causes are so far unknown and it is difficult to compare studies with different vaccine production procedures.

There are no or very few field scientific studies using control groups [12,13] demonstrating that autogenous vaccines, produced by licensed companies, are able to induce an immune (antibody) response and/or whether or not their use is correlated with a clear reduction in both mortality and antibiotic use. In fact, it is unknown if this practice, as presently used, is economically profitable for producers. In addition, there is no clear information on how these vaccines should be applied. In the field, even in the absence of scientific studies, autogenous vaccines are sometimes given to young piglets without any data about their capacity to induce an immune response, and most likely in the presence of maternal antibodies. On the other hand, many producers prefer vaccination of sows before farrowing which, in theory, would elicit passive maternal immunity. The latter protocol is less costly, and thus it represents an attractive economical alternative to piglet vaccination. However, very few studies have addressed how long this maternal immunity lasts in piglets [14], especially considering that most clinical cases caused by *S. suis* take place in the post-weaning period. 

In the present study, the humoral immune response (levels, isotypes and killing capacity of generated antibodies) and protective effect of an autogenous vaccine produced by a licensed company and given to either piglets or sows was scientifically evaluated in the field.

## 2. Materials and Methods 

### 2.1. Selected Farm and Clinical History

A 3700-sow farrow-to-finish system in Canada with internal gilt replacement and no commingling was selected. The farm was considered to be “positive stable” for PRRSV based on the definition of Holtkamp et al. [15]. Piglets were weaned at 3 weeks of age into different single source sites. The farm had recurrent *S. suis* problems in all nursery sites, with clinical cases beginning mostly at 5 weeks of age, with spikes between 6 and 10 weeks of age. No *S. suis*-like clinical signs were observed in suckling piglets. Total mortality in nursery varied between 2.5% and 4%, where many clinical cases were due to *S. suis*. Indeed, the latter was kept lower than expected due to prophylactic and therapeutic use of antibiotics. Antibiotic susceptibility testing was routinely performed by a private diagnostic service and confirmed that the strains were susceptible to penicillin, amoxicillin, ampicillin, ceftiofur, florfenicol, tiamulin and trimethoprim/sulfadoxine. The medication used to treat animals with clinical signs associated to *S. suis* infections was benzylpenicillin procaine or ceftiofur. Samples (meningeal swabs, joint swabs and spleen) from different nursery piglets were submitted at multiple occasions for complete diagnosis. Serotyping of *S. suis* isolates from these samples was carried out by the same laboratory responsible for the autogenous vaccine production as previously described [16]. Although a final diagnosis of *S. suis* serotype 7-related diseases was given, during the project, a second serotype (serotype 9) was also identified as being responsible for several clinical cases (see below). Piglets were also vaccinated against porcine circovirus, *Mycoplasma hyopneumoniae* and PRRSV (CircoFlex/MycoFlex/PRRS^®^, Boehringer Ingelheim, Ingelheim am Rhein, Germany) at weaning and again 3 weeks later against *M. hyopneumoniae* (Respisure One^®^, Zoetis, Parsippany, NJ, USA). 

### 2.2. Vaccine Preparation

Autogenous vaccine was prepared by a company possessing the official license for manufacturing such products (i.e., authorized by the local authorities to produce the vaccine). It was composed of *S. suis* serotype 7 strain 1718 (isolated from the brain of a piglet with meningitis) for the first experiment (piglet vaccination), and an additional *S. suis* serotype 9 strain 117827-21 (isolated from the spleen of a clinical case of sudden death during the first experiment) was added for the second experiment (sow vaccination). Both vaccines were combined with a strain of *Staphylococcus hyicus* and a strain of *Glaeserella parasuis*. An oil-in-water emulsion adjuvant (confidential formulation) was used to prepare the autogenous vaccine. No ethical statement was required for this study, as the protocol used was part of normal interventions in the farm and performed by the veterinarian in charge, as stated by the Animal Welfare Committee of the University of Montreal.

### 2.3. Immunization Protocol 

**Figure 1:**
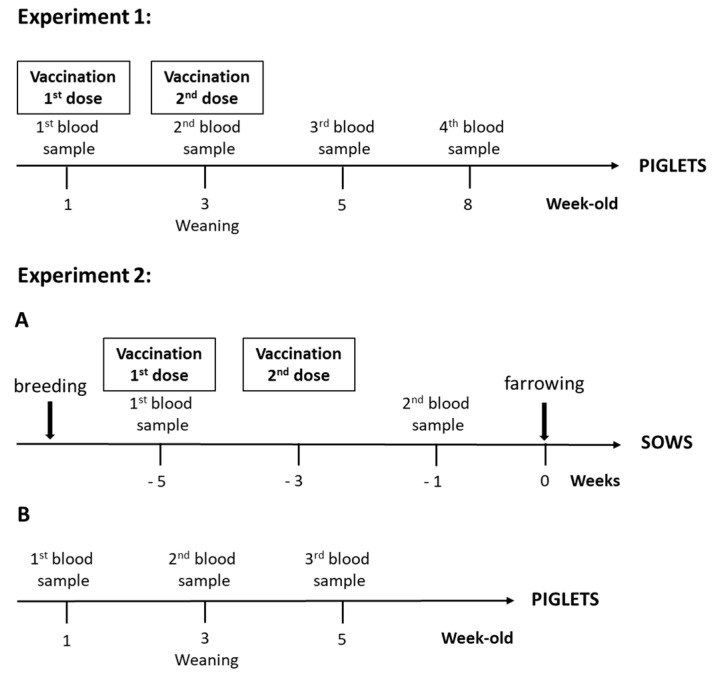
Experimental design of the field study. Experiment 1: Piglets from non-vaccinated sows were vaccinated during the first week at 4 days ± 2 and at weaning with the autogenous vaccine (containing *S. suis* serotype 7). Blood samples were collected from randomly chosen piglets at 1, 3, 5 and 8 weeks of age. Control (including placebo animals) were also included. Experiment 2: (**A**) Sows were at their parity 0 and received two doses of autogenous vaccine (containing *S. suis* serotypes 7 and 9) intramuscularly at 5 and 3 weeks before farrowing. Blood samples were taken from all enrolled sows prior to vaccination at 5 weeks before farrowing and at 1 week before farrowing (2 weeks after the 2nd vaccine dose). (**B**) Randomly selected piglets from vaccinated and non-vaccinated sows were sampled at 1, 3 and 5 weeks of age.

For the first experiment (Figure 1), piglets from non-vaccinated sows were selected (*n* = 1494). During the first week, at 4 days ± 2, farm staff randomly divided each litter into two groups. Group 1 received the autogenous vaccine intramuscularly in the neck plus an ear notch (“vaccinated group”; *n* = 583) and Group 2 was processed normally as per farm procedures (“non-vaccinated group”; *n* = 911). Within this latter group, 20 randomly selected animals were injected with a placebo solution, with adjuvant only. All animals used in our experiments were identified with an ear tag. Each litter was roughly divided equally into each group. Piglets from Group 1 received a second dose vaccine at weaning (three weeks of age). Group 2 did not receive any injection at weaning, except for the 20 piglets injected with the placebo. Farrowing piglets over the course of 1 week were enrolled in the trial as described above. All piglets were randomly weaned into three nursery rooms in the same barn, with pigs from Groups 1 and 2 mixed in each pen. Water-soluble medication was withheld for 21 days post-weaning.

For the second experiment (Figure 1), vaccinated and non-vaccinated parity 0 sows were randomly selected (*n* = 20/group). Sows received two doses of the autogenous vaccine intramuscularly at 5 and 3 weeks before farrowing. In total, 207 piglets from vaccinated sows and 183 piglets from non-vaccinated sows were enrolled in the trial. All piglets were weaned into one nursery room, with mixed vaccinated and non-vaccinated animals in each pen. Water-soluble medication was withheld for 21 days post-weaning.

### 2.4. Blood Sampling

For the first experiment (Figure 1), blood samples were collected from randomly chosen tagged piglets at 1, 3, 5 and 8 weeks of age from 50 vaccinated and 70 non-vaccinated animals, including the 20 that received a placebo, to follow the immune response. 

For the second experiment (Figure 1), blood samples were taken from all enrolled sows at 5 weeks (before vaccination) and 1 week (after 2-dose vaccination) pre-farrowing. In total, 120 of the randomly selected and tagged piglets (3 piglets/sow, for a total of 60 piglets/group) from vaccinated and non-vaccinated sows were originally included in the serological study. However, nine piglets from three different non-vaccinated sows could not be identified, resulting in 51 piglets being sampled in this group. Piglets were sampled at approximately 1, 3 and 5 weeks of age.

Collected sera from both experiments were stored at −20 °C until analyzed by ELISA and for the opsonophagocytosis assay (described below).

### 2.5. Enzyme-Linked Immunosorbent Assay (ELISA) for Pig Immunoglobulin (Ig) Titers

Strains used in the autogenous vaccine were also used for the coating of ELISA Polysorb plates (Nunc-Immuno; Thermo Scientific, Mississauga, ON, Canada) [17,18]. Bacteria were grown overnight onto 5% sheep blood agar plates at 37 °C, and isolated colonies were cultured in 5 mL of Todd–Hewitt broth (THB) (Becton Dickinson, Mississauga, ON, Canada) for 8 h at 37 °C with agitation at 120 rpm. Then, 10 μL of 1/1000 dilution of 8-h cultures were transferred into 30 mL of THB and incubated for 16 h at 37 °C with agitation at 120 rpm. Stationary-phase bacteria were washed in phosphate-buffered saline (PBS) at pH 7.3. Bacterial pellet was then suspended in ddH_2_O and adjusted to a concentration equivalent at 10^8^ CFU/mL. Plates were coated with 100 μL/well of the whole bacterial suspension, air-dried during two days at room-temperature (RT) and finally fixed with 50 μL/well of 100% methanol. After evaporation of methanol, plates were stored at RT until use. For titration of antibodies, plates were washed with PBS–Tween (PBS-T) containing 0.05% Tween 20 and blocked for 1 h with 2% skim milk in PBS-T at RT. After washing, 100 µL of different 2-fold-based dilutions of pig sera (in PBS-T) were added to each well and incubated for 1 h at RT. For titration of porcine total Ig [IgG + IgM] or IgM, plates were incubated with peroxidase-conjugated goat anti-pig total Ig [IgG + IgM] (Jackson ImmunoResearch, West Grove, PA, USA) or IgM (AbD Serotec, Raleigh, NC, USA) antibodies for 1 h at RT. For porcine IgG1 or IgG2 detection, mouse anti-porcine IgG1 or IgG2 (BioRad) was added for 1 h at RT. After washing, peroxidase-conjugated goat anti-mouse IgG (Jackson ImmunoResearch, West Grove, PA, USA) was added for 1 h at RT. Plates were developed with 3,3′,5,5′-tetramethylbenzidine (TMB; Invitrogen, Burlington, ON, Canada) substrate, and the enzyme reaction was stopped by addition of 0.5 M H_2_SO_4_. Absorbance was read at 450 nm with an ELISA plate reader. The reciprocal of the last serum dilution that resulted in an optical density at 450 nm (OD_450_) of ≤ 0.2 (cutoff) was considered the titer of that serum. To control inter-plate variations, an internal reference positive control was added to each plate. This positive control was composed by a pool of serum of six sows randomly selected in the farm that showed high ELISA values against *S. suis* serotype 7 and serotype 9 because of their natural exposition to these serotypes in the farm. Reaction in TMB was stopped when an OD_450_ of 1.0 was obtained for the positive internal control. Optimal dilutions of the positive internal control sera and anti-porcine antibodies or conjugates were determined during preliminary standardizations.

### 2.6. ELISA for Pig Antibodies against Serotype 7 S. suis Capsular Polysaccharide (CPS)

In selected experiments, to measure anti-CPS specific antibodies, 1000 ng of purified native *S. suis* serotype 7 CPS in PBS (pH 7.4) were added to wells of ELISA Polysorb plates (Nunc-Immuno) following a previously standardized protocol [17]. Purified CPS antigen was produced as previously described [19]. After overnight coating at 4 °C, plates were washed with PBS-T and blocked for 1 h with 2% skim milk in PBS-T at RT. After washing, 100 µL of different 2-fold-based dilutions of pig sera (in PBS-T) were added to each well and incubated for 1 h at RT. After washing, the plates were incubated with peroxidase-conjugated antibodies as described above.

### 2.7. Opsonophagocytosis Assay (OPA) 

The OPA test was based on that described for mice [17] but adapted to swine sera. Whole blood, as a source of total phagocytic cells, was obtained from young naive piglets originating from a farm without *S. suis* endemic infection and intravenously collected in vacutainer sodium heparin tubes (Becton Dickinson, Franklin Lakes, NJ, USA), and kept at RT. Using washed bacterial cultures grown as described above, final bacterial suspensions were prepared in complete cell culture medium (RPMI 1640 supplemented with 5% heat-inactivated fetal bovine serum, 10 mM HEPES, 2 mM L-glutamine and 50 μM 2-mercaptoethanol; Invitrogen) to obtain a concentration of 2 × 10^6^ CFU/mL. The number of CFU/mL in the final suspension was determined by plating samples onto THB agar (THA). Whole blood (containing approximately 1 × 10^8^ leukocytes/mL) was mixed with the *S. suis* suspension to obtain a multiplicity of infection (MOI) of 0.01. Control and sample sera from immunized animals were added to a concentration of 40% *v*/*v* in microtubes to a final volume of 200 μL. Control sera came from naïve pigs (absorbed against *S. suis* serotype 7 and presenting negative ELISA values), and positive sera were obtained and pooled from sows (originated from the same farm and presenting high ELISA values). The tube tops were pierced using a sterile needle and were incubated for 4 h at 37 °C with 5% CO_2_, with gentle agitation. After incubation, viable bacterial counts were performed on THA using an Autoplate 4000 automated spiral plater. The percentage of bacterial killing was determined using the following Formula (1):

% Bacteria killed = [1 − (bacteria recovered from sample tubes/bacteria recovered from negative control tube with control serum)] × 100(1)

### 2.8. Clinical Evaluation of Animals

For both experiments, clinical signs, mortality and injectable treatments were recorded from all enrolled piglets by farm staff daily by room. Pigs were identified as notched or not notched, and there were no individual pig identifiers. As such, an individual pig could be represented more than once in the data set for treatments. Pigs were followed until the end of the nursery period (10 weeks of age). When possible, meningeal swabs or other tissue samples were collected from pig mortalities and submitted for culture and *S. suis* serotyping. Isolation was performed by culture in blood agar and identification of *S. suis*-like colonies by PCR [20]. Identified *S. suis* isolates were further serotyped by PCR [21]. 

### 2.9. Statistical Analyses

ELISA data were log-10 transformed to normalize distributions. Unless otherwise specified, a linear mixed model was used with sampling time as the within-subject fixed effect, group (vaccinated or not vaccinated) as the between-subject fixed effect, and animal identification (id) as random effect. For Experiment 1, piglet id was used. For Experiment 2, sow id was used for sow serology analyses; in the case of piglets, sow id and piglet id nested within sow were used as random effects. The model also took into account unequal variances in the two groups. A priori contrasts were performed to compare pairs of means adjusting the alpha level downward for each comparison with the sequential Benjamini–Hochberg procedure. In the analysis of IgG1 and IgG2 subclasses in sow sera (Experiment 2) or anti-CPS antibodies in piglet sera (Experiment 1), equal variance *t*-test was used to compare means according to status. For OPA analyses, data were arcsine square-root transformed to normalize distributions. For the association between ELISA titers and OPA test (percent of killing), data were normalized as described above. A covariance analysis with the titers as a cofactor and the group as the independent variable was performed. The model allowed us to test the hypothesis that there was a linear relationship between the titers and the percent of killing (OPA test) as well as to evaluate if this relationship was similar between groups. Statistical analyses were performed using SAS 9.4 (SAS, Cary, NC, USA). The level of statistical significance was set at 0.05. 

## 3. Results

### 3.1. Experiment 1: Piglet Vaccination

#### 3.1.1. An Active Vaccination Program of Piglets with an Autogenous Bacterin Fails to Increase Antibody Levels Post-Weaning

**Figure 2:**
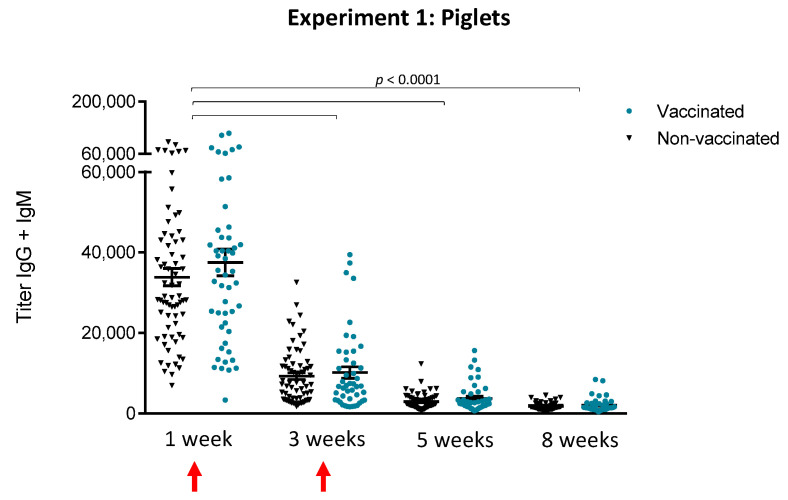
Experiment 1: Kinetics of total Ig against *S. suis* serotype 7 in piglets. Blood samples were collected from randomly chosen (and tagged) piglets at one, three, five and eight weeks of age from 50 vaccinated and 70 non-vaccinated (including the 20 placebos) animals to follow the immune response. The vaccination protocol is shown in Figure 1. Total Ig [IgG + IgM] titers were determined by ELISA. Antibody titers for individual piglets are shown with horizontal bars representing mean ± SEM. Values significantly different are shown in the graph with corresponding *p* value. Arrows indicate first and second vaccination doses.

The kinetics of total Ig [IgG + IgM], using *S. suis* serotype 7 whole bacteria as antigen, induced by a two-dose vaccination program applied to piglets was quantitatively evaluated (Figure 2). As expected, no differences were observed between animals that received adjuvant only and other non-vaccinated animals in the control group (*p* > 0.05). Therefore, results from these animals were grouped together for all subsequent analyses. No statistically significant differences were observed between vaccinated and non-vaccinated animals at any time point evaluated (Figure 2). Interestingly, antibody levels were very high before vaccination in the first week, probably of maternal origin. However, levels were significantly (*p* < 0.0001) lower at three weeks of age and continued to decrease until five weeks of age, at the moment of the onset of *S. suis*-related clinical signs (see Figure 5 in Section 3.1.4). Vaccination of piglets failed to increase their antibody levels or duration. 

#### 3.1.2. The Isotype Profile of the Antibody Response in Piglets Was Unchanged by Vaccination with the Autogenous Bacterin

**Figure 3:**
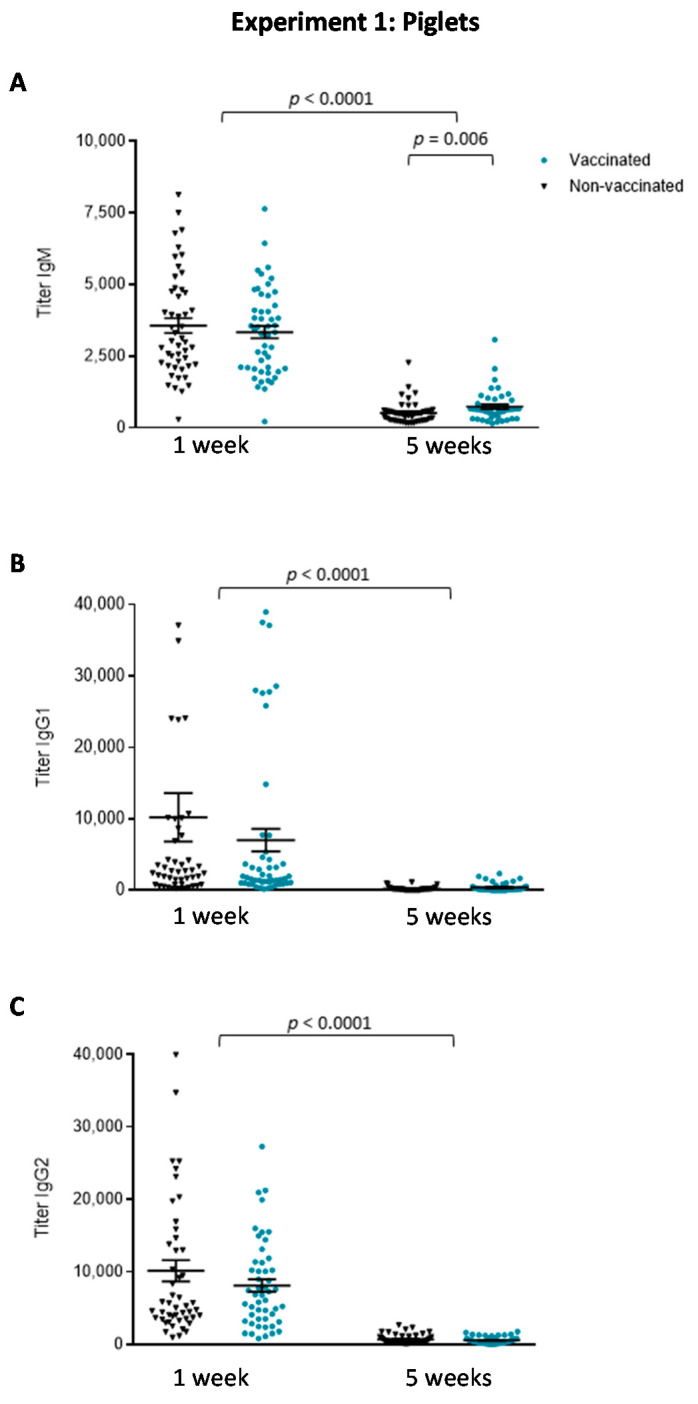
Experiment 1: Isotype profile of antibodies against *S. suis* serotype 7 in piglets. Blood samples were collected from randomly chosen (and tagged) piglets at one and five weeks of age from 50 vaccinated and 50 non-vaccinated (including the 20 placebos) animals to evaluate: (**A**) IgM titers; (**B**) IgG1 titers; and (**C**) IgG2 titers by ELISA. The vaccination protocol is shown in Figure 1. Antibody titers for individual piglets are shown with horizontal bars representing mean ± SEM. Values significantly different are shown in the graph with corresponding *p* value.

Analysis of total Ig [IgG + IgM] antibodies (using the vaccine strain of *S. suis* serotype 7) does not accurately discriminate differences in individual Ig isotypes. To this aim, serum samples obtained before vaccination and at five weeks of age (after the second vaccination) were used to quantify levels of IgM, IgG1 and IgG2 (Figure 3). Before vaccination, the antibodies present in the sera, probably of maternal origin, were mainly composed of IgG1 and IgG2 isotypes, with lower levels of IgM. At five weeks of age, the level of all isotypes dropped independently of vaccination, when compared to levels at one week (*p* < 0.0001). Overall, no differences were observed in the Ig isotype profile between vaccinated and non-vaccinated piglets. Only titers of IgM were slightly, but significantly, higher in vaccinated than in non-vaccinated animals at five weeks of age (*p* = 0.006). 

#### 3.1.3. An Active Vaccination Program of Piglets with an Autogenous Bacterin Fails to Improve the Killing Capacity of Antibodies

**Figure 4:**
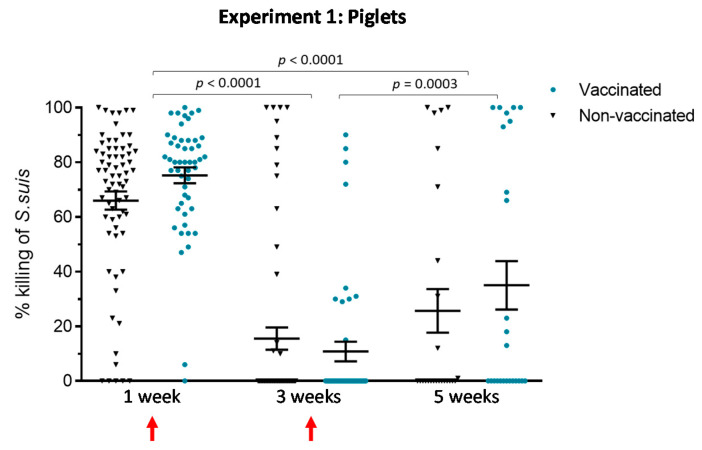
Experiment 1: Opsonophagocytosis killing of *S. suis* serotype 7 induced by serum antibodies from piglets. Blood samples were collected from randomly chosen piglets at one, three and five weeks of age. Blood samples from 50 vaccinated and 70 non-vaccinated (including the 20 placebos) at one and three weeks of age and from 25 vaccinated and 25 non-vaccinated (including the 20 placebos) at five weeks of age were tested in an opsonophagocytosis assay (OPA) to evaluate their functionality. For OPA, blood leukocytes were mixed with *S. suis* serotype 7 (vaccine strain) at a multiplicity of infection of 0.01. Control sera or sample sera from vaccinated piglets or non-vaccinated piglets were added to a final concentration of 40% *v*/*v* in microtubes which were incubated for 4 h. After incubation, viable bacterial counts were performed and the percentage of bacterial killing was determined. The results are expressed as percent of bacterial killing for individual sera, with horizontal bars representing mean ± SEM. Values significantly different are shown in the graph with corresponding *p* value. Arrows indicate first and second vaccination doses.

In vaccine studies, in vitro functional assays, such as the opsonophagocytosis assay (OPA test), complement ELISA titers for evaluating protection, and they are largely used in human medicine as a correlate of immunity (e.g., for pneumococcal conjugate vaccine). The OPA test evaluates the capacity of vaccine-induced antibodies to kill bacteria in the presence of phagocytic cells. Such assays are normally performed using phagocytic cell lines or purified cell types, which underestimates the complexity of blood bactericidal activity. In this study, we standardized an OPA test using swine whole-blood as effector cells, in a format that requires small serum quantities. After incubation, viable bacterial counts are performed. The percent of bacterial killing is determined by comparing bacterial numbers in sample tubes with those in negative control tubes. As such, the positive control used was considered showing 100% of killing. Sera at one and two weeks after the first vaccine dose (three weeks of age) and two weeks after the second vaccine dose (five weeks of age) were evaluated in the OPA test. The results show no statistically significant differences between vaccinated and non-vaccinated animals at any time point evaluated (Figure 4). Nevertheless, within the vaccinated group, animals having received their second dose (five weeks of age) showed a slightly higher OPA activity than those having their first dose (three weeks of age) (*p* = 0.0003). Interestingly, there was a statistically significant correlation (Figure S1; *p* = 0.03) between IgM levels (Figure 3) and percent of killing at five weeks of age within the vaccinated group. 

Noticeably, before vaccination, maternal-derived antibodies were already highly opsonizing and thus able to induce bacterial elimination by phagocytic cells. However, the OPA activity of these antibodies was significantly reduced at three and five weeks of age (*p* < 0.0001), with no differences between vaccinated and control animals. 

#### 3.1.4. Clinical Outcome Was Not Improved by an Active Vaccination Program of Piglets with an Autogenous Bacterin

Mortality and injectable treatments were recorded by farm staff daily in the three nursery rooms (total of 1494 piglets; 583 vaccinated and 911 non-vaccinated). As shown in Table 1, overall mortality rate was 6.0%, with a rate of 5.3% in the vaccinated group and of the 6.5% in the non-vaccinated group. The incidence rate of total injectable treatments was 0.73% in vaccinated animals and of the 0.89% in non-vaccinated ones (Table 2). 

**Figure 5:**
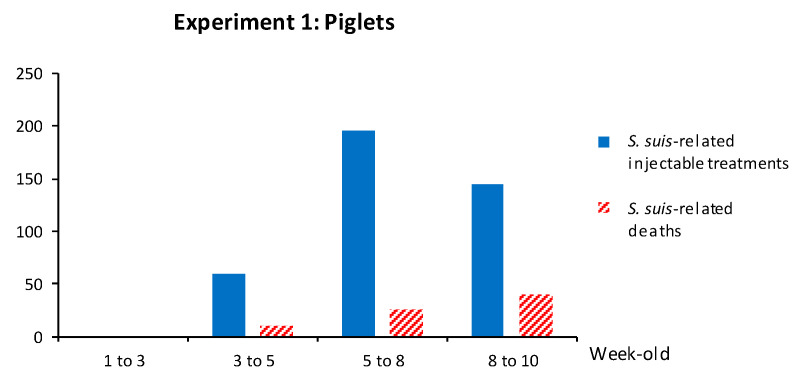
Experiment 1: Number of *S. suis*-related injectable treatments and *S. suis*-induced number of deaths in piglets over time. Piglets from non-vaccinated sows were selected (*n* = 1494). During the first week, at 4 ± 2 days, each litter was randomly divided into two groups. Group 1 (“vaccinated group”; *n* = 583) received the autogenous vaccine (as shown in Figure 1) and Group 2 was processed normally (“non-vaccinated group”; *n* = 911). Piglets were randomly weaned into three nursery rooms, with pigs from Groups 1 and 2 mixed in each pen. *S. suis*-related mortality and injectable treatments were recorded from all enrolled piglets by farm staff daily by room. Pigs were identified as ear notched or not, and there were no individual pig identifiers. As such, an individual pig could be represented more than once in the dataset for treatments. Pigs were followed until the end of the nursery period at approximately 10 weeks of age. In this graph, only *S. suis*-related mortality and injectable treatments are displayed.

Other than mortality due to sudden death, clinical signs related to *S. suis* were mostly associated to arthritis and meningitis. The kinetics of *S. suis*-related mortality and *S. suis*-related injectable treatments (as described in Section 2) showed that clinical disease started at five weeks of age (Figure 5). Mortality gradually increased until eight weeks of age. *S. suis*-related mortality was 4.8% in vaccinated animals and 5.5% in non-vaccinated animals (Table 1). In total, 12 isolates of *S. suis* were recovered from diseased animals, and nine of them were serotype 7. Unexpectedly, three isolates belonged to serotype 9, which was not previously detected during the diagnostic procedure carried out prior to the autogenous vaccine production. One of these isolates was then included in the autogenous vaccine used in sows (see below). It is important to note that a systematic epidemiological survey could not be performed to determine the relative frequencies of these serotypes in clinical cases, since samples from all cases were not sent to the laboratory.

### 3.2. Experiment 2: Sow Vaccination

#### 3.2.1. Antibody Levels against the Autogenous Vaccine Are Increased in Vaccinated Sows, yet the Ig Isotype Profile Differs between the Serotypes

**Figure 6:**
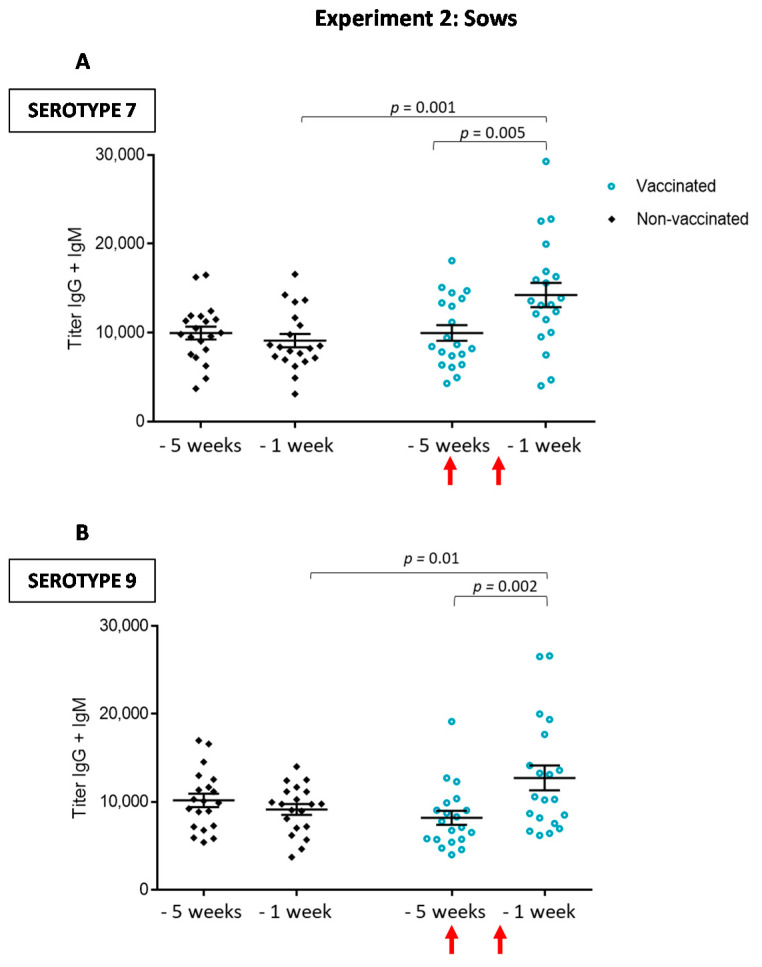
Experiment 2: Total Ig levels against *S. suis* serotype 7 (**A**) or serotype 9 (**B**) in sows. Blood samples were collected one and five weeks before farrowing from 20 vaccinated and 20 non-vaccinated sows to follow the immune response. The vaccine was administered at five and three weeks before farrowing. Total Ig [IgG + IgM] titers were determined by ELISA. Antibody titers for individual sows are shown with horizontal bars representing mean ± SEM. Values significantly different are shown in the graph with corresponding *p* value. Arrows indicate first and second vaccination doses.

Due to the finding showing that *S. suis* serotype 9 was also clinically important in the herd, the producer decided to include both serotype 7 and serotype 9 strains in the *S. suis* autogenous bacterin used for the two-dose vaccination program of sows (Experiment 2, Figure 1). Levels of total Ig [IgG + IgM] against *S. suis* serotype 7 and *S. suis* serotype 9 were already very high before vaccination (Figure 6). Interestingly, titers significantly increased after the second vaccine dose, as measured one week before farrowing (Figure 6). 

**Figure 7:**
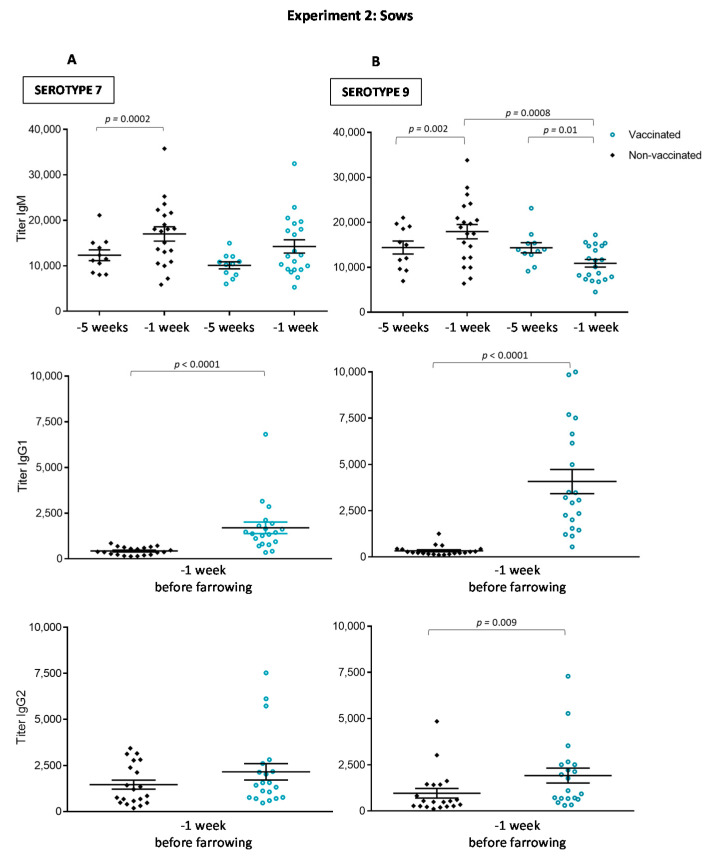
Experiment 2: Isotype profile of antibodies against *S. suis* serotype 7 (**A**) or serotype 9 (**B**) in sows. Blood samples were collected one and five weeks before farrowing from 20 vaccinated and 20 non-vaccinated sows to follow the immune response. The vaccination protocol is shown in Figure 1. IgM, IgG1 and IgG2 titers were determined by ELISA. Antibody titers for individual sows are shown with horizontal bars representing mean ± SEM. Values significantly different are shown in the graph with corresponding *p* value.

An effective vaccine is expected to induce not only a higher anti-*S. suis* Ig levels but also isotype switching from IgM to IgG, as normally evidenced by a reduction in IgM and an increase in IgG subclasses during a secondary immune response (Figure 7). This was clearly observed for serotype 9 (Figure 7B), as vaccinated sows presented a reduced titer of IgM and increased levels of IgG. Both IgG1 and IgG2 subclasses against serotype 9 were higher in vaccinated sows compared to non-vaccinated controls; however, IgG1 was the most dominant antibody subclass. Isotype switching against serotype 7 was less evident, with only a significant increase in IgG1 observed in vaccinated animals compared to controls (Figure 7A). Finally, in the control group, IgM levels were significantly higher at −1 week than at −5 weeks before farrowing (Figure 7). This natural increase in IgM was probably the result of continuous exposition of animals to *S. suis* or cross-reacting bacteria from microflora, including other streptococci.

#### 3.2.2. Maternal Antibody Transfer to Piglets Is Not Increased after Sow Vaccination

**Figure 8:**
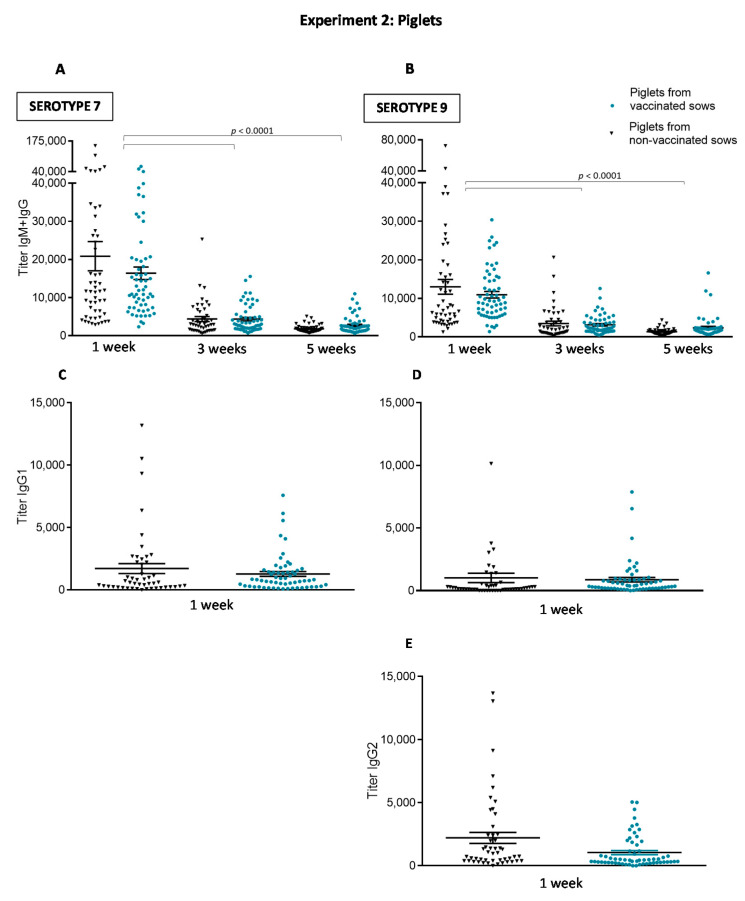
Experiment 2: Kinetics of total Ig and isotype profile of antibodies against *S. suis* serotype 7 or serotype 9 in piglets from either vaccinated or non-vaccinated sows. Randomly selected 120 piglets (3 piglets/sow, for a total of 60 piglets/group) from vaccinated and non-vaccinated sows were originally included in the serological study. Nevertheless, nine piglets from three different non-vaccinated sows could not be identified, for a total of 51 piglets finally enrolled for serology in this group. Piglets were sampled at one, three and five weeks of age and total Ig [IgG + IgM] titers were determined by ELISA against *S. suis* serotype 7 (**A**) or serotype 9 (**B**). Piglets were sampled at one week to evaluate titers of IgG1 against *S. suis* serotype 7 (**C**) or serotype 9 (**D**) and titers of IgG2 against *S. suis* serotype 9 (**E**). Antibody titers for individual piglets are shown with horizontal bars representing mean ± SEM. Values significantly different are shown in the graph with corresponding *p* value.

**Figure 9:**
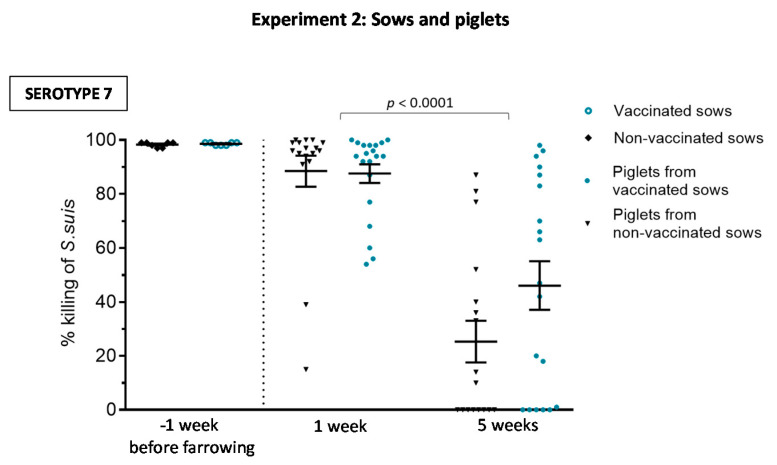
Experiment 2: Opsonophagocytosis killing of *S. suis* serotype 7 induced by serum antibodies from sows and from their piglets. Blood samples were collected one week before farrowing from seven vaccinated and seven non-vaccinated sows and from randomly chosen piglets (1 per sow) at one and five weeks of age (*n* = 20 per group) to evaluate their functionality in an opsonophagocytosis assay (OPA). For OPA, blood leukocytes were mixed with *S. suis* serotype 7 (vaccine strain) at a multiplicity of infection of 0.01. Control sera or sample sera from vaccinated or non-vaccinated animals were added to a final concentration of 40% *v*/*v* in microtubes, which were incubated for 4 h. After incubation, viable bacterial counts were performed and the percentage of bacterial killing determined. The results are expressed as percent of bacterial killing for individual sera, with horizontal bars representing mean ± SEM. Values significantly different are shown in the graph with corresponding *p* value.

The goal of sow vaccination is to induce an increase of maternal antibody transfer to piglets through colostrum intake. Despite higher levels of anti-*S. suis* Ig [IgG + IgM] induced in sows by the autogenous bacterin, piglets from vaccinated sows showed similar levels of antibodies against either serotype 7 or serotype 9 than piglets from non-vaccinated sows at one week (Figure 8A,B). Vaccination of sows did not improve duration of maternal antibodies in piglets; titers were already low at weaning. Since IgG1 and IgG2 subclasses against serotype 9 and IgG1 subclass against serotype 7 were significantly increased in vaccinated sows, we analyzed if these particular IgG subclasses were also predominantly transferred to piglets. However, isotype profile of piglets from vaccinated sows was similar to those from non-vaccinated sows (Figure 8C–E). Finally, the functional ability of the antibodies was also evaluated using serotype 7 as a model and for comparative purposes with the first part of the study. As shown in Figure 9, the OPA activity of antibodies in sows one week before farrowing was 100% in both groups. Due to maternal transfer of these functionally active antibodies, OPA capacity was also high in the sera of piglets at one week, with no differences between piglets derived from vaccinated vs. non-vaccinated sows (Figure 9; *p* = 0.76). As observed in Experiment 1 (Figure 4), OPA activity of antibodies was significantly lower at five weeks of age. At this age, levels were similar in both groups (*p* = 0.041). 

#### 3.2.3. Weaned Piglets Are Not Clinically Protected after a Sow Vaccination Program with Two Doses of the *S. suis* Autogenous Bacterin

**Figure 10:**
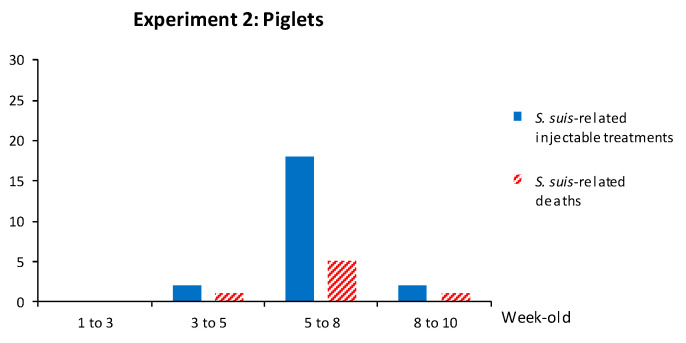
Experiment 2: Number of *S. suis*-related injectable treatments and *S. suis*-related deaths in piglets over time. In total, 207 piglets from vaccinated sows and 183 piglets from non-vaccinated sows were enrolled in the trial. Piglets were weaned into one nursery room, with mixed vaccinated and non-vaccinated groups in each pen. Total and *S. suis*-related mortality and injectable treatments were recorded from all enrolled piglets by farm staff daily by room. Pigs were identified as notched or not notched, and there were no individual pig identifiers. As such, an individual pig could be represented more than once in the dataset for treatments. Pigs were followed until the end of the nursery period. In this graph, only *S. suis*-related mortality and injectable treatments are displayed.

In total, 207 piglets from vaccinated sows and 183 piglets from non-vaccinated sows were followed for clinical outcomes and injectable treatments were recorded. The mortality rate related to *S. suis* disease was 3.3%: 3.4% in the vaccinated group and 3.3% in the non-vaccinated group (Table 3). Mortality and clinical signs related to *S. suis* were similar to those described above. The kinetics of *S. suis*-related mortality and *S. suis*-related injectable treatments (such as penicillin G) showed that clinical disease peaked at five weeks of age (Figure 10). When bacterial isolation and serotyping were possible, either serotype 7 or serotype 9 (*n* = 6) was found in brain and/or joint swabs of clinically affected animals.

### 3.3. The Autogenous Vaccine Induces the Production of Anti-CPS Antibodies in Sows but Not in Piglets after Vaccination

**Figure 11:**
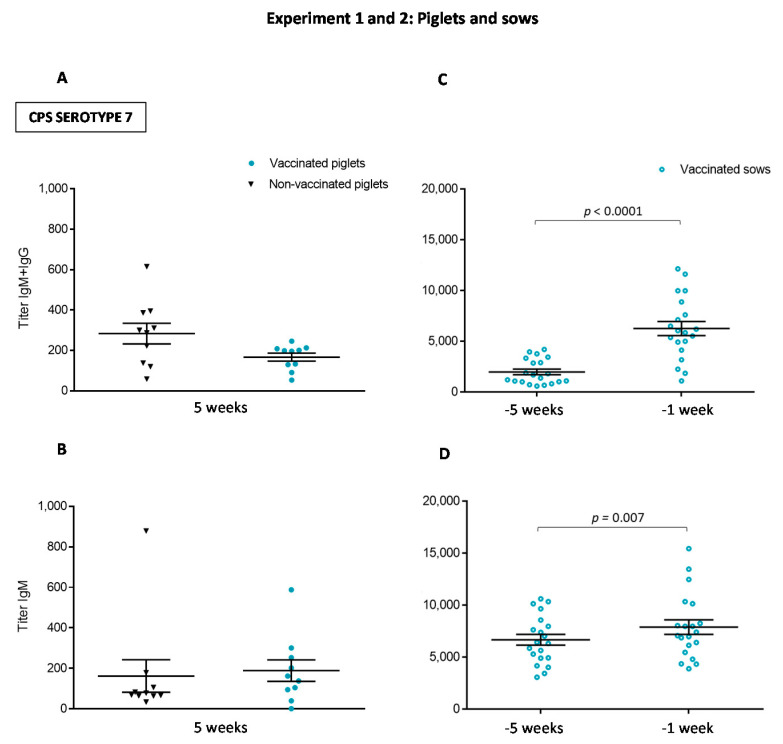
Experiment 1 and 2: Total Ig and IgM profile of antibodies against *S. suis* serotype 7 purified capsular polysaccharide (CPS) from either vaccinated or non-vaccinated piglets from Experiment 1 (**A**,**B**) and vaccinated sows from Experiment 2 (**C**,**D**). Randomly selected 20 piglets (10 vaccinated and 10 non-vaccinated) from Experiment 1 and 20 randomly selected vaccinated sows from Experiment 2 were used for the quantification of antibodies against *S. suis* serotype 7 CPS. Total Ig [IgG + IgM] titers (**A**,**C**) and IgM titers (**B**,**D**) were determined by ELISA against purified *S. suis* serotype 7 CPS. Piglets were sampled at five weeks to evaluate titers of antibodies against serotype 7 CPS and sows were sampled at one and five weeks before parturition. Antibody titers for individual piglets or sows are shown with horizontal bars representing mean ± SEM. Values significantly different are shown in the graph with corresponding *p* value.

The CPS is an important virulence factor of *S. suis*, and antibodies against the CPS are thought to be highly protective [7]. However, the ability of bacterins to induce this type of antibodies is controversial [7,17]. To evaluate the capacity of the autogenous vaccine used herein, an anti-CPS ELISA was performed. For the first experiment, 20 piglets (10/group) were randomly selected for the quantification of total Ig [IgG + IgM] and IgM against serotype 7 CPS (Figure 11A,B). Serum samples were collected at five weeks of age for this quantification, therefore after two vaccine doses. For the second experiment, 20 vaccinated sows were randomly selected (Figure 11C,D). Serum samples were collected at one and five weeks before farrowing, i.e., prior to vaccination (basal levels) and after two vaccine doses. Sow antibody titers against the serotype 7 CPS, either total Ig [IgG + IgM] or IgM, were higher compared to those present in piglets. After vaccination, piglets from Experiment 1 did not generate an increase in antibody titers against the CPS (Figure 11A,B). However, sows from Experiment 2 showed a significant increase of both total Ig [IgG + IgM] titers (*p* < 0.0001) and IgM titers (*p* = 0.007) after vaccination (Figure 11C,D). 

## 4. Discussion

In the era of antibiotic restrictions, new social consumption trends and lack of efficacious commercial vaccines, autogenous bacterins dominate the market with the intention to control *S. suis* infection in pigs. It is important to clearly differentiate “experimental bacterins” from “licensed autogenous vaccines”. Experimental bacterins are usually produced by research laboratories using well characterized (and usually reference) strains and powerful adjuvants, some of them no longer available [22]. In the field, only companies possessing the official license for manufacturing autogenous vaccines can produce them, using authorized adjuvants. To the best of our knowledge, only two published articles address the efficacy of this preventive approach in the field with inclusion of control groups and using a licensed (and not experimental) vaccine. In the recent study of Hopkins et al. [12], vaccine effectiveness was evaluated on a farrow-to-finish farm experiencing mortality due to *S. suis*. Direct, indirect, total and overall vaccine effectiveness were analyzed by vaccinating 75% of pigs in each litter. The selected pigs received the autogenous *S. suis* serotype 2 vaccine, at weaning and a booster three weeks after entering the nursery. The direct effect of vaccination (mortality due to *S. suis*) was not statistically significant. However, the calculated total and overall vaccine effectiveness (complete herd-level mortality) showed potential protective effects; however, mortality due to any cause was considered here. In the study of Torremorel et al. [13], piglets were vaccinated at weaning and boosted 10 days later and mortality and morbidity rates in nursery pigs fluctuated regardless of treatment. These results are in agreement with our clinical data and suggest that successful control of *S. suis*-related disease by an autogenous vaccination program remains to be more carefully assessed. The vaccination strategy might have an impact on vaccine effectiveness, including the time of piglet prime and boost vaccination or the number of required doses in sows according to parity. The dynamics of *S. suis* infection in the herd might also affect the conclusions that can be driven from field studies, as suggested in previous published works [12,13]. In our study, the emergence or misplacing of serotype 9 in the piglet vaccination program could have affected the vaccine capacity to reduce *S. suis*-related clinical signs in the farm. Indeed, serotype 9 might have been on the farm since the beginning but was not detected earlier (i.e., not detected during the first diagnostic screening), which reflects the importance of the diagnostic procedures before implementing an autogenous vaccine. 

Another important confounding factor is the assessment of total mortality or total treatments instead of those directly related to clinical signs compatible with *S. suis* disease. If an outbreak of an unrelated disease was present on the farm and further controlled during the vaccination trial, the improvement in health might not be directly linked to the vaccine effect. The opposite, such as a concomitant infection causing piglet mortality, might also negatively affect the evaluation of the autogenous vaccine. In our study, total morbidity/mortality and total injectable treatments were carefully recorded. *S. suis*-related disease or treatment was estimated based on clinical sings. However, it should also be taken into consideration that confirmatory necropsy, followed by bacteriology and *S. suis* serotyping were not systematically performed in all clinical cases. Although no cases of polyserositis were identified, clinical cases due to *G. parasuis* were previously identified in the farm and some clinical signs are common between the two pathogens [1]. Finally, the use of preventive medication, which differs from farm to farm, also complicates the analysis of clinical data, by masking the real effect of the vaccine and/or keeping *S. suis*-related mortality levels very low and thus compromising statistical power. The inclusion of control groups in the study design (when possible) helps reducing the impact of these misleading factors; nevertheless, interpretation of clinical outcomes after vaccination remains challenging in field studies. Despite these acknowledged limitations of field studies, gathered information is vital to improve cost-effective implementation of this important vaccine strategy. 

An important aspect of vaccine evaluation is the study of the underlying immunological responses induced by the vaccine that can be scientifically and directly linked to vaccine effectiveness. Indeed, our study is the first to characterize the antibody response in terms of magnitude, kinetics, isotype profiling and functionality; all key aspects in vaccinology. *S. suis* is an encapsulated pathogen and its CPS protects the bacterium against immune system clearance by phagocytic cells, thus allowing *S. suis* systemic dissemination [23,24,25]. This natural resistance of *S. suis* is overcome if highly opsonic antibodies recognizing surface-exposed bacterial components, or the CPS itself, are present These antibodies will induce rapid bacterial uptake by phagocytic cells and consequent destruction [17]. As such, when evaluating the efficacy of a *S. suis* vaccination program, it is also vital to analyze the OPA activity of vaccine-induced antibodies. 

In this study, an active vaccination program of piglets failed to induce a significant increase in anti-*S. suis* antibody levels, or changes in the isotype profile as evaluated by ELISA, or functionality as measured by the OPA test. Only a small increase in IgM levels was observed after boost vaccination of piglets. At this time point (five weeks of age), an increase in OPA activity was also observed compared to that observed after prime vaccination. However, no overall differences with the placebo group were observed in the OPA test. Albeit promising, the biological implication of an increase in these parameters was limited as no clinical protection was observed at this time point, which coincided with an increase of *S. suis*-related disease in the nursery. 

The immunological characterization of the antibody response at approximately one week of age (before piglet vaccination) also revealed impressively high levels of antibodies, most probably of maternal origin, and this independently of sow vaccination. This response was mainly composed, as expected for maternal antibodies, of IgG1 and IgG2 and lower levels of IgM. This maternal antibody response was highly opsonic, yet not lasting enough to confer protection in the post-weaning period. Indeed, at the moment of appearance of *S. suis* clinical signs, antibody levels in piglets were already very low, independently of the vaccination program used. Similar to our findings, an experimental study showed that neither application of *S. suis* bacterin to preparturient sows nor that to suckling piglets or both elicited protection in eight-week-old piglets, which was explained by the lack of opsonizing antibodies. Serum half-life for IgG in suckling piglets was estimated around 6–10 days [14,26]. A previously published serological cross-sectional profile of unvaccinated piglets also showed a significant decrease in anti-*S. suis* antibody levels after two weeks of age, with the lowest values occurring between six and eight weeks [27].

Based on these observations, a high maternal interference when vaccinating piglets during the first two weeks of age could be expected. In a previous field study using an experimental bacterin [27], the response to the vaccine varied considerably among pigs and was attributed, at least in part, to the levels of maternal antibodies at the time of vaccination. In the experimental study of Baums et al. [14], the missing or weak immune responses observed with regard to suckling piglet vaccination (two doses at two and four weeks of age) was potentially related to either inhibition by maternal antibodies and other colostrum components or immature adaptive immunity in suckling piglets. Therefore, more research is needed to evaluate the perfect age window for piglet vaccination in order to avoid maternal interference but confer protection at the moment of *S. suis* clinical signs onset. 

The immunological characterization of the antibody response during the sow vaccination experiment notably revealed that basal antibody levels against *S. suis* in sows are already very high. This was also noticed in previous studies [14,27]. These antibodies are probably generated by either natural exposition of animals to *S. suis* on the farm, especially taking into account that this bacterial species is part of the normal microflora of pigs, or by cross-reactions with other commensal bacterial species, including other streptococci. A two-dose vaccination program of sows before farrowing significantly increased anti-*S. suis* antibody levels, with isotype switching towards IgG1 mainly. However, this increased response in sows was not sufficient to significantly improve maternal antibody transfer to piglets and thus their clinical protection over time. Interestingly, although levels of maternal antibodies of piglets from either vaccinated of non-vaccinated sows were very high during the first week of age, an important variation in the antibody titers (with a few piglets presenting very low values) was observed. This was also previously noticed in another study [27]. Possible explanations may include lack of sufficient colostrum intake or intestinal absorption problems in those piglets. 

The OPA activity of sow antibodies was already 100% before vaccination and thus no changes can be expected by a vaccination program of sows. The high levels of sow antibodies, as detected by ELISA, and high level of opsonic antibodies, as measured by the OPA test, may explain the usual lack of clinical disease in adult animals [1]. In addition, despite increased Ig titer levels in sows, OPA activity of maternal-derived antibodies in piglets from vaccinated sows was similar to that observed in the control group. Overall, the vaccination program applied to sows in our study failed to improve pre-existing natural protective immunity and its functionality in piglets. Sow vaccination programs are highly variable, which can affect the vaccine response and remains to be fully evaluated in a field comparative study.

The isotype profile of vaccine-induced antibodies has been reported to be important when evaluating protection against *S. suis*, as this would be linked to the capacity of certain isotypes to induce opsonophagocytosis while other isotypes are supposed to be poorly opsonic [28,29]. In this study, the results show that IgM naturally increased, probably as the result of continuous exposition of animals to *S. suis* or cross-reacting bacteria from microflora, as aforementioned. Regarding isotype switching, the adjuvant is the main component of the vaccine formulation driving isotype switch and this aspect has not been scientifically evaluated in field vaccination with autogenous bacterins. This may be explained by the use of vaccines prepared by licensed private companies, which do not usually disclose the formulation. The vaccine formulation used in this study seems to favor, at least in sows, production of IgG1 subclass, which has been previously suggested to be poorly protective in the context of *S. suis* infections [28]. The vaccine antigen itself can also influence the isotype switching and/or antibody functionality [30,31]. In this study, isotype switching was less efficiently induced by the serotype 7 compared to the serotype 9 vaccine-component. As such, the impact of autogenous vaccine formulation on its efficacy needs to be carefully studied, including the effect of the adjuvant and the possible interference by inclusion of multiple *S. suis* serotypes and strains, as it is commonly used in the field. 

As aforesaid, the CPS is a major virulence factor of *S. suis* and an important target of protective antibodies [7,17]. Nevertheless, due to its polysaccharide nature, the CPS is a low immunogenic antigen when compared to proteins. CPS antigens normally induce low memory responses and limited isotype switch (i.e., the response is mainly composed of IgM) [32]. The capacity of bacterins to induce anti-CPS antibodies has been poorly studied and only under experimental conditions. These few studies reported contradictory results. Vaccination trials of weaned piglets with an experimental *S. suis* serotype 2-bacterin adjuvanted with Stimune^®^ (Prionics, La Vista, NE, USA) either failed to induce a significant increase of total Ig levels of anti-CPS antibodies [17] or induced an increase in anti-CPS total antibody titers, yet at much lower levels than those induced against whole bacteria [22]. In another study, weaned piglets vaccinated with a *S. suis* serotype 2-bacterin formulated with Emulsigen^®^ (Phibro Animal Health Corporation, Teaneck, NJ, USA) adjuvant did not show significant levels of anti-CPS Ig [IgG + IgM] antibodies [33]. When a similar formulation was used to vaccinate sows at three and five weeks antepartum, an increase in levels of anti-CPS antibodies was observed [14]. Herein, using an autogenous vaccine applied in the field, levels of total Ig as well as IgM against the CPS of serotype 7 also increased in sows vaccinated at three and five weeks antepartum. However, the piglet vaccination program failed to induce an increase of anti-CPS antibodies. It should be noted that basal levels of anti-CPS antibodies were already high in sows prior to vaccination, as observed for antibodies against the whole bacteria. This feature, once again, supports the hypothesis that adult animals are immune against *S. suis* and, coincidentally, clinical cases are almost never observed in adult animals. Overall, it seems that bacterins possess a weak capacity to induce anti-CPS antibodies in piglets, which might be affected by the age of animals, time of vaccination, vaccine preparation and formulation (mainly the adjuvant), as well as probably the targeted *S. suis* serotype. 

## 5. Conclusions

Autogenous bacterins are presently the only preventive tool available for swine producers, as an alternative to antimicrobials, to control *S. suis* infections. This promising approach requires extensive and comparative scientifically-sound studies to evaluate the most efficacious way to prepare the vaccine, the adjuvant to be included, the number of doses, age of piglet vaccination and the real benefit of vaccinating sows, or piglets or both. Finally, it is important to remember that the overall efficacy of autogenous vaccines cannot be determined based on results obtained with one particular batch of vaccine prepared by a single licensed laboratory. Methods used for the vaccine production, bacterial concentration and the adjuvant used (among other variables) may highly influence the results obtained.

## Figures and Tables

**Table 1:** Distribution of mortality among vaccinated and non-vaccinated piglets.

Experiment 1 ^1^	All Deaths		*S. suis*-Related Deaths		Alive	Total Pigs
Vaccinated	31	5.32%	28	4.80%	552	583
Non-vaccinated	59	6.48%	50	5.50%	852	911
Total pigs	90	6.02%	78	5.22%	1404	1494

^1^ Vaccinated (*n* = 583) and non-vaccinated (*n* = 911) piglets were followed clinically. *S. suis*-related death rate was calculated recognizing limits without autopsy.

**Table 2:** Distribution of treatment data among vaccinated and non-vaccinated piglets in the post- weaning period.

Experiment 1 ^1^	Number of Injectable Treatments	Time in Pig Weeks (No. Pigs in Each Group × No. Days in Nursery)	Incidence Rate of Treatments
Vaccinated	208	28,567	0.73%
Non-vaccinated	398	44,639	0.89%
Total treatments	606	73,206	0.83%

^1^ Vaccinated (*n* = 583) and non-vaccinated (*n* = 911) piglets were followed clinically. The number of injectable treatments given to sick pigs was recorded. One pig could have received more than one injection. No., number.

**Table 3:** Distribution of mortality among piglets from vaccinated and non-vaccinated sows.

Experiment 2 ^1^	*S. suis*-Related Deaths		Alive	Total Pigs
Vaccinated	7	3.38%	200	207
Non-vaccinated	6	3.28%	177	183
Total pigs	13	3.33%	377	390

^1^ Piglets from vaccinated sows (*n* = 207) and piglets from non-vaccinated sows (*n* = 183) were followed clinically. *S. suis*-related death rate was calculated recognizing limits without autopsy.

## Supplementary Materials

The following are available online at https://www.mdpi.com/2076-393X/8/3/384/s1, Figure S1: Correlation between IgM levels and % of killing at 5 weeks of age in the vaccinated piglet group. Blood samples were collected from randomly chosen (and tagged) piglets at 5 weeks of age from 50 vaccinated animals to evaluate IgM titers and opsonophagocytosis assay (OPA) activity. A *p* value < 0.05 indicates a positive association.
