# Supplementary material for: Field Study on the Immunological Response and Protective Effect of a Licensed Autogenous Vaccine to Control Streptococcus suis Infections in Post-Weaned Piglets
Source: Vaccines (Basel). 2020 Jul 14;8(3):384. doi: 10.3390/vaccines8030384 (PMC7565864; doi:10.3390/vaccines8030384)
Supplement: Supplementary file 1 [file vaccines-08-00384-s001.pdf]

Supplementary data

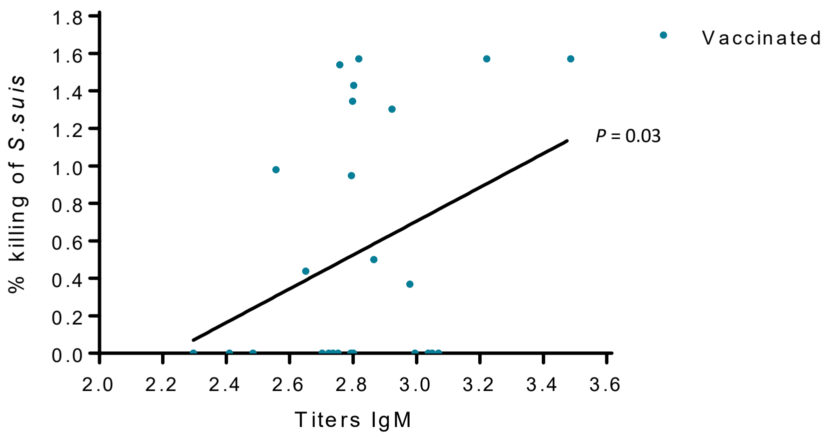

Figure S1. Experiment 1: Correlation between IgM levels and % of killing at 5 weeks of age in the vaccinated piglet group. Blood samples were collected from randomly chosen (and tagged) piglets at 5 weeks of age from 50 vaccinated animals to evaluate IgM titers and opsonophagocytosis assay (OPA) activity. A *P* value < 0.05 indicates a positive association.
